# Computational Design of Apolipoprotein E4 Inhibitors for Alzheimer's Disease Therapy from Traditional Chinese Medicine

**DOI:** 10.1155/2014/452625

**Published:** 2014-05-21

**Authors:** Hung-Jin Huang, Hsin-Yi Chen, Cheng-Chun Lee, Calvin Yu-Chian Chen

**Affiliations:** ^1^Department of Chinese Pharmaceutical Sciences and Chinese Medicine Resources, College of Pharmacy, China Medical University, Taichung 40402, Taiwan; ^2^Department of Biomedical Informatics, Asia University, Taichung 41354, Taiwan; ^3^School of Medicine, College of Medicine, China Medical University, Taichung 40402, Taiwan

## Abstract

Apolipoprotein E4 (Apo E4) is the major genetic risk factor in the causation of Alzheimer's disease (AD). In this study we utilize virtual screening of the world's largest traditional Chinese medicine (TCM) database and investigate potential compounds for the inhibition of ApoE4. We present the top three TCM candidates: Solapalmitine, Isodesacetyluvaricin, and Budmunchiamine L5 for further investigation. Dynamics analysis and molecular dynamics (MD) simulation were used to simulate protein-ligand complexes for observing the interactions and protein variations. Budmunchiamine L5 did not have the highest score from virtual screening; however, the dynamics pose is similar to the initial docking pose after MD simulation. Trajectory analysis reveals that Budmunchiamine L5 was stable over all simulation times. The migration distance of Budmunchiamine L5 illustrates that docked ligands are not variable from the initial docked site. Interestingly, Arg158 was observed to form H-bonds with Budmunchiamine L5 in the docking pose and MD snapshot, which indicates that the TCM compounds could stably bind to ApoE4. Our results show that Budmunchiamine L5 has good absorption, blood brain barrier (BBB) penetration, and less toxicity according to absorption, distribution, metabolism, excretion, and toxicity (ADMET) prediction and could, therefore, be safely used for developing novel ApoE4 inhibitors.

## 1. Introduction

Alzheimer's disease (AD) is the most common harmful neurological disorder affecting patients over the age of 65 [1]. The major neuropathological hallmarks of AD are neurofibrillary tangles and beta amyloid plaques in the entorhinal cortex and hippocampus [2]. Deposition of *β*-amyloid is associated with neuronal loss and cognitive dysfunction [3], which affects the ability to study and think. There is also long-term memory loss during the life span. *β*-amyloid is generated from cleavage of the amyloid precursor protein (APP) by *β*-secretase and *γ*-secretase [4–8]. Several genes linked to familial AD have been identified in many studies, but there are still more risk-factor genes that remain to be identified [9]. The variations of apolipoprotein E (*AOPE*) genes as potential genetic risk factors for AD have been identified by the linkage studies of Pericak-Vance et al. [10]. The ApoE gene is located on chromosome 19q13.2, and an increase in type 4 allele of this gene is associated with several chronic neurodegenerative diseases [11].

ApoE, which exists in three different isoforms in the brain and periphery, ApoE2 (epsilon 2), ApoE3 (epsilon 3), and ApoE4 (epsilon 4), have an essential role in the regulation of cholesterol metabolism [12–14]. In human blood circulation, ApoE binds lipids and makes them soluble for transporting [13]. In the nervous system, ApoE transports cholesterol to neurons in the brain [15]. ApoE4 is the most important known genetic risk factor in the causation of sporadic AD [16, 17], as further studies have confirmed [18], due to ApoE4 binding to *β*-amyloid, and transporting it to neurons. The human allele frequency of ApoE4 in Caucasian patients with AD is 36.7% [19].

We utilized computer-aided drug design (CADD) in this research in order to design potential lead drugs for AD therapy. CADD is an efficient approach for the rapid identification of potential lead compounds in target therapy [20, 21] and has been widely used in drug design including virus therapy [22–26], cancer therapy [27–31], treatment of sleeplessness [32], neuropathic therapy [33–38], weight loss therapy [39, 40], diabetic treatment [41], inflammation treatment [42], erectile dysfunction treatment [43], and hair loss therapy [44]. For target proteins, drug design should depend on some illness research [25, 41, 45–49], risk-factor studies [50–53], web server [23, 54], and scientific theories [55]; mutant proteins are also important factors as drug targets [56]. Traditional Chinese medicine (TCM) was first developed in China and has been used more than 2000 years in Asian countries. In this research, we utilize small TCM compounds from the world's largest TCM database (TCM Database@Taiwan) [57] to investigate potential lead drugs for ApoE4. The database virtual screen provides rapid search to identify potent compounds for ApoE4 interactions. Molecular dynamics (MD) simulation is used to simulate the dynamic change between ApoE4 protein and docked ligand.

## 2. Materials and Methods

### 2.1. Data Sets

We used 61,000 TCM compounds for database screening, which were obtained from TCM Database@Taiwan [57], with the drug-likeness of all compounds being predicted by ADMET prediction. The crystal structure of ApoE4 was taken from PDB database (PDB code:1GS9), the missing atoms and loops were cleaned up by* Prepare Protein module* under Accelrys Discovery Studio 2.5.5.9350 (DS 2.5) [58], and all residues were protonated under pH 7.4 conditions. We also employed disorder predict tool (PONDR-FIT) [59] to predict unfolded regions on ApoE4 sequence for structure validation.

### 2.2. Docking Analysis

The LibDock program [60] of DS 2.5 was used to define protein site features referred to polar and nonpolar features, with a sphere of 35 Å radius used as the binding area. Different rigid ligand conformations were placed into the binding area, and all ligand conformations were minimized using the CHARMm force field. Minimization performed 1000 steps of Steepest Descent with a RMS gradient tolerance of 3, which was then followed by the Conjugate Gradient. The generated ligands were docked into the defined binding site on the ApoE4 protein structure. Ligand binding in the receptor cavity was evaluated by the scoring functions of the LibDock score. Ligplot plus was used to analysis docking poses for H-bond and hydrophobic interactions [61, 62].

### 2.3. Molecular Dynamics Simulation

The molecular dynamic simulation was performed with GROMACS 4.5.5 package [63] for protein-ligand complexes simulation and the charmm27 force field was used in the simulation system. For box definition, distance of real space cut-off was set to 1.2 nm. The particle mesh Ewald (PME) method was regarded as coulomb type for treating electrostatics, and the cut-off distance of defining van der Waals (VDW) residues was set at 1.4 nm. In pair potentials versus many-body potentials [64–67], the potential functions representing the nonbonded energy of VDW and electrostatics using the following:

(1) $$\begin{array}{c} U\left(r\right)=4\varepsilon \left[{\left(\frac{\delta }{\gamma }\right)}^{12}-{\left(\frac{\delta }{\gamma }\right)}^{6}\right],\\ U_{ij}\left(r_{ij}\right)=\sum \frac{Z_{i}Z_{j}}{4\pi \varepsilon 0}\frac{1}{r_{ij}}+\sum A_{l}\exp\frac{-r_{ij}}{Pl}+\sum C_{l}r_{ij}^{-nl}. \end{array}$$

All bonds were constrained with the linear constraint solver (LINCS) algorithm for fixing all bond lengths. The TIP3P model was employed for water simulation. Topology files and parameters of small compounds in protein-ligand complexes were generated for GROMACS simulation by the SwissParam web server; the concentration of NaCl model was set as 0.145 M in the solvent system. The Steepest Descent algorithm performed the 5,000 cycle steps used for energy minimization. This was followed by equilibration performed under position restraints for 1 ns under constant temperature dynamics (NVT type) conditions. Following this step, all production dynamics simulations were performed for 5000 ps under constant pressure and temperature dynamics (NPT type). The temperature in all of the simulation systems was set as 310 K. MD conformations were saved every 20 ps for trajectory analysis.

## 3. Results and Discussion

### 3.1. Docking Analysis

**Figure 1:**
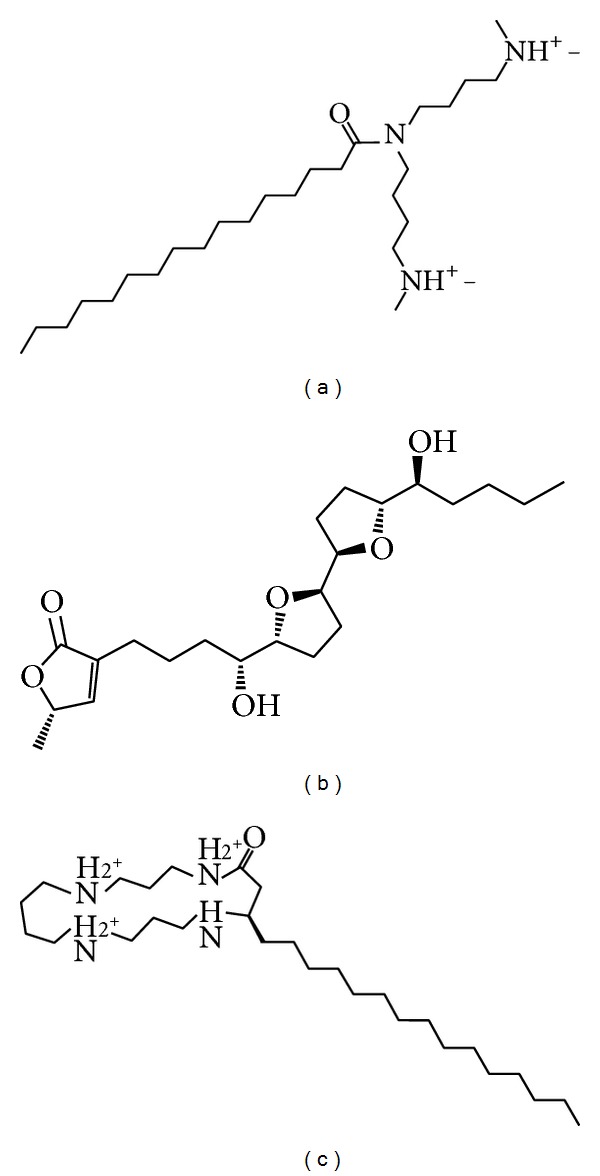
Chemical scaffold of (a) Solapalmitine, (b) Isodesacetyluvaricin, and (c) Budmunchiamine L5.

**Figure 2:**
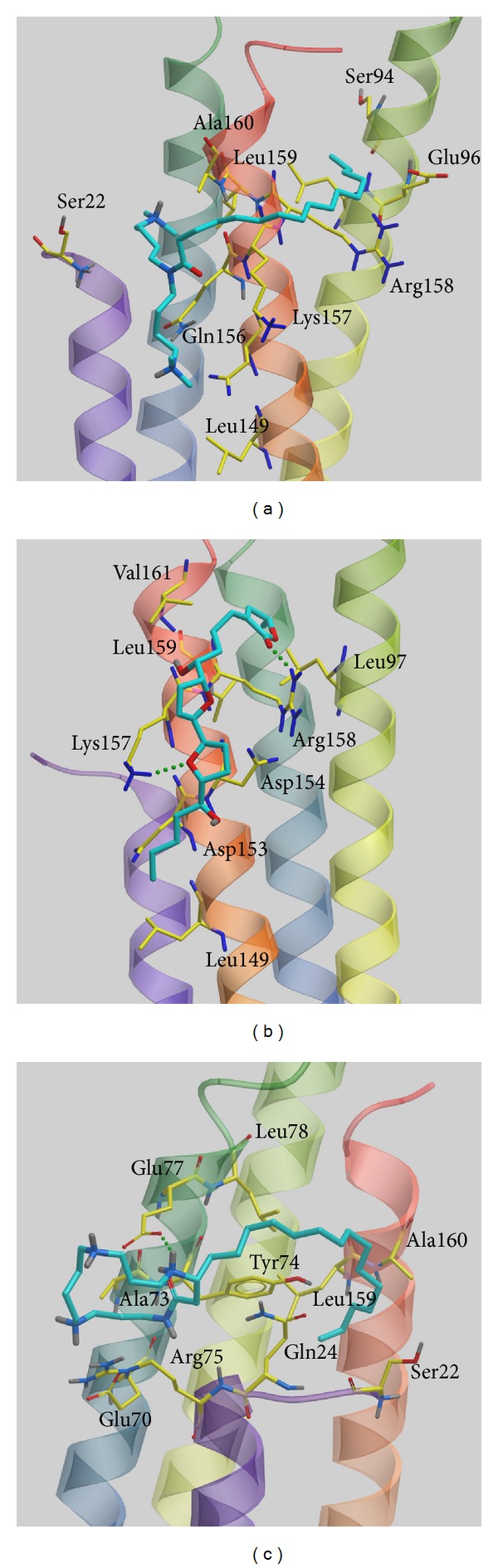
Docking poses of top three candidates: (a) Solapalmitine, (b) Isodesacetyluvaricin, and (c) Budmunchiamine L5. The small molecular and amino acids are colored in green and yellow, respectively.

**Figure 3:**
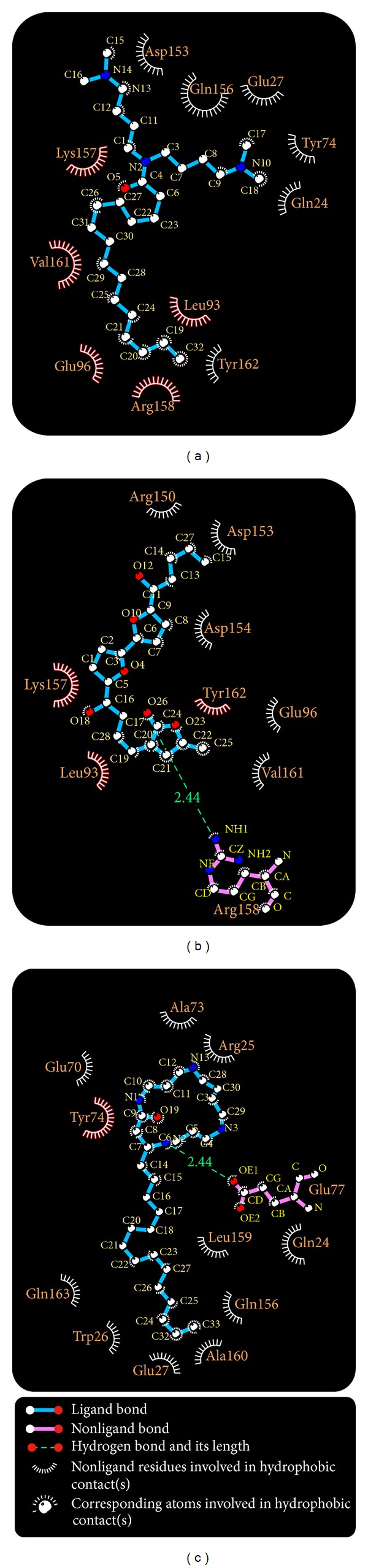
H-bond and hydrophobic analysis of docking poses by Ligplot plus tool for each docked ligand in ApoE4: (a) Solapalmitine, (b) Isodesacetyluvaricin, and (c) Budmunchiamine L5.

We used the LibDock score to select potent TCM compounds which have high affinity with ApoE4. The results of the docking score are listed in Table 1 and all candidates are ranked by LibDock Score. We also used ADMET prediction to evaluate the drug-likeness of TCM candidates. All TCM candidates had a good absorption prediction for metabolism. In toxicity evaluation no TCM candidate displayed CYP2D6 inhibiting and hepatotoxicity, suggesting that these ligands have no toxicity in the liver. Blood brain barrier (BBB) penetration showed that these ligands have good penetration and may be suitable for central nerve system therapy. We regard the top three TCM candidates, Solapalmitine, Isodesacetyluvaricin, and Budmunchiamine L5, as potential compounds and further analyze the binding poses in ApoE4 protein structure. Scaffold of the top three TCM compounds is shown in Figure 1, and the docking poses of each ligand are displayed in Figure 2. For H-bond analysis, Solapalmitine had no H-bonds with any residue of ApoE4. The docking pose of Isodesacetyluvaricin revealed H-bonds with Lys157 and ARG158. For Budmunchiamine L5, the docked ligand formed two H-bonds with Glu77. In the 2D diagram of docking poses by Ligplot (Figure 3), Solapalmitine did not reveal any H-bonds, while the surrounding residues, Asp153, Gln156, Gln27, Tyr74, Gln24, Lys157, Val161, Leu93, Tyr162, Glu96, and Arg158, displayed hydrophobic interactions with the ligand. The 2D diagram of Isodesacetyluvaricin was similar to the 3D docking poses, which form H-bond with Arg158; hydrophobic interaction residues included Arg150, Asp153, Asp154, Tyr162, GLu96, Val161, Lys157, Leu93, and Arg158. For Budmunchiamine L5, the 2D diagram is corrected with 3D docking pose, and only Glu77 can form H-bonds with the docked ligand; this ligand also interacted with Ala73, Arg25, Glu70, Tyr74, Glu77, Gln24, Leu159, Gln156, Ala160, Glu27, Trp26, and Gln163 by hydrophobic forces.

**Figure 4:**
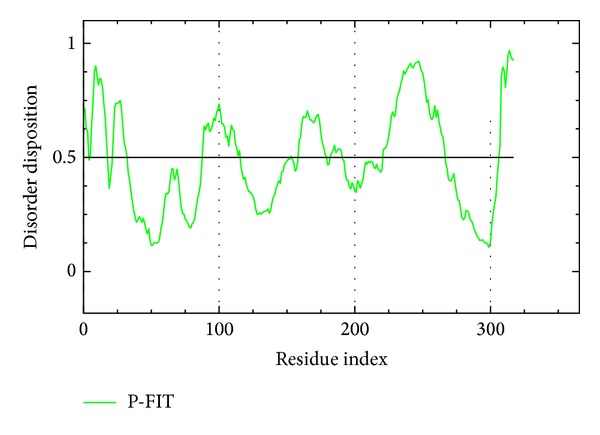
Disorder prediction of sequence of ApoE4 from the results of PONDR-FIT. The value of disorder disposition above 0.5 in disorder disposition.

In comparison with the docking study, PONDR-FIT was used to predict the disorder region among all residues on ApoE4 (Figure 4). According to the drug design of protein structure [68], ordered folding regions do not influence ligand binding [69, 70]. Most of binding residues for the top three compounds were below 0.5, suggesting that the structure of ApoE4 will not affect ligands binding to the active site. To further analyze the variation of the docked protein structure, we utilized MD simulation to generate a dynamic structure for binding analysis of the top three TCM compounds.

### 3.2. Stability Analysis

**Figure 5:**
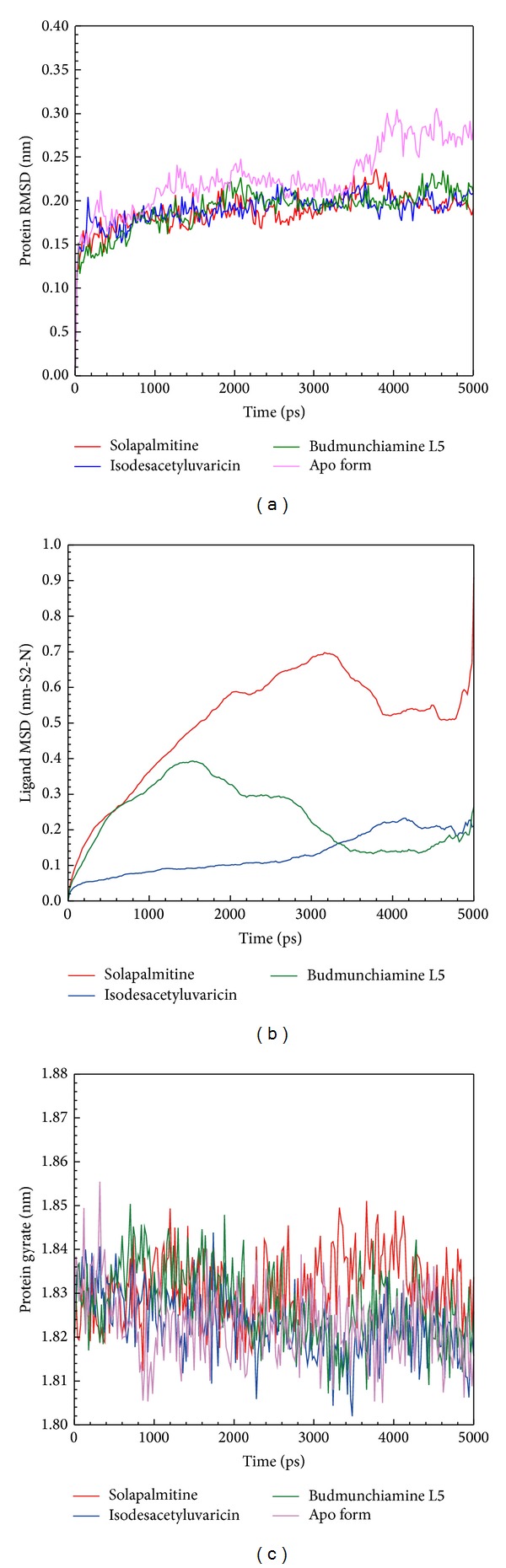
Plots of (a) protein RMSD, (b) ligand MSD, and (c) radius of gyration from ApoE4 with docked ligand or no ligand (apo) with a simulation time of 5000 ps.

**Figure 6:**
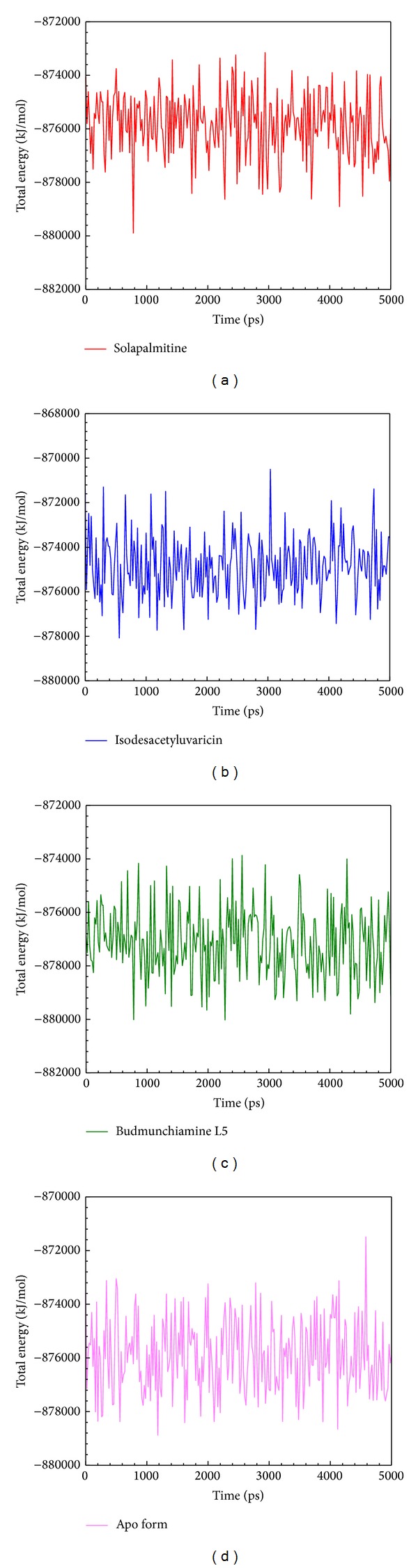
Total energy of ApoE4 with docked ligand: (a) Solapalmitine, (b) Isodesacetyluvaricin, and (c) Budmunchiamine L5 from all simulation times; the no-ligand binding protein (d) was used as the control.

Complexes of ApoE4 with docked ligands were performed by MD simulation at 5000 ps, and ApoE4 with no ligand (Apo protein) were regarded as the control for comparison. Each plot of the root mean square deviation (RMSD), mean square displacement (MSD), and radius of gyration (*R*_*g*_) is displayed in Figure 5. The Apo protein of ApoE4 changed significantly from 0.20 to 0.27 nm after 4000 ps, indicating that ApoE4 with ligand was more stable during MD simulation. The MSD analysis was used to calculate the migration distance among all simulation times. The MSD values for Budmunchiamine L5 increased from 1500 to 5000 ps, which displayed a similar movement distance to Isodesacetyluvaricin at final simulation time. Solapalmitine had an increased MSD value with a simulation time of 5000 ps, suggesting that Solapalmitine is less stable than the other two TCM candidates. The gyration assay was used to analyze the compactness of protein structure. *R*_*g*_ values of all protein-ligand complexes and Apo protein had similar fluctuations, indicating all structures tended to become stable after MD simulation. For total energy analysis, no significantly increased values were observed among all simulation times (Figure 6). The total energy of all systems remained in −876000 kJ/mol. These results suggest that all structures of the complexes tend to become constant after the initial simulation time.

### 3.3. Residues Fluctuation and Distance Analysis

**Figure 7:**
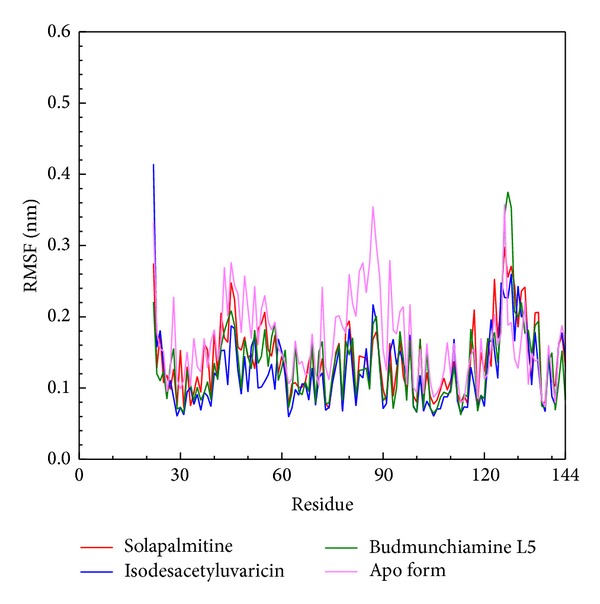
RMSF values of ApoE4 with docked ligand or no ligand (Apo) (a) Solapalmitine, (b) Isodesacetyluvaricin, and (c) Budmunchiamine L5 with simulation times of 5000 ps; the no-ligand binding protein (d) was used as the control.

**Figure 8:**
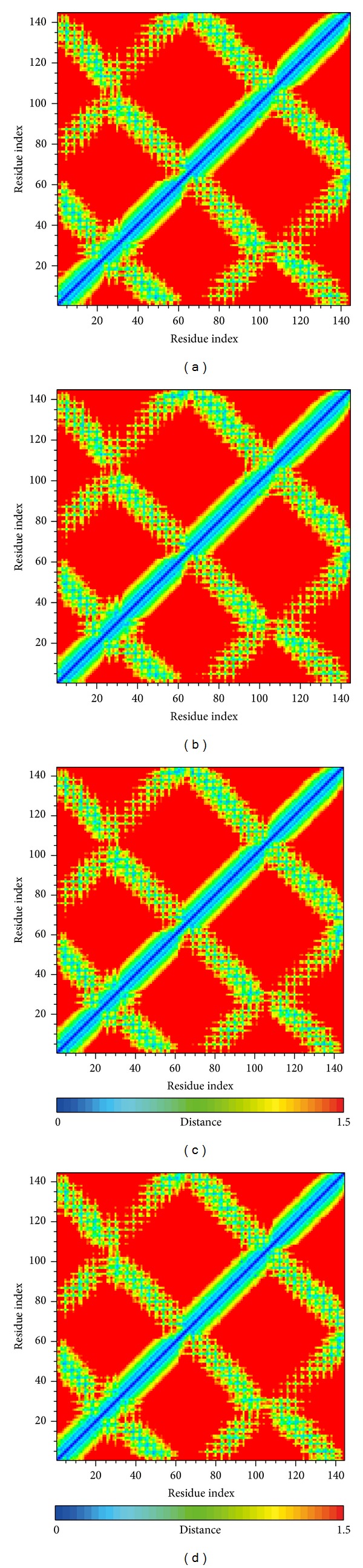
Matrix of smallest distance between each pair of amino acids in the complex with (a) Solapalmitine, (b) Isodesacetyluvaricin, and (c) Budmunchiamine L5; the no-ligand binding protein (d) is used as the control.

Root mean squared fluctuation (RMSF) was carried out to analyze the fluctuation of residues on ApoE4 protein (Figure 7). It is obvious that residues of Apo protein from 70 to 100 exhibit substantial fluctuation, but the three candidates remain stable. The ligand binding region is included in this region, but the docked residues are not flexible due to the largest fluctuations being exhibited at terminal residues, and these regions are far from the docked residues. The results suggest that the docked ligand could bind stably to ApoE4. The matrices of distance maps for residue-residue distance calculations over 5000 ps are shown in Figure 8. The results display that all complexes with docked ligands are the same as Apo protein, suggesting that the conformations do change among all MD simulations.

### 3.4. Clustering Analysis for Snapshot Selection

**Figure 9:**
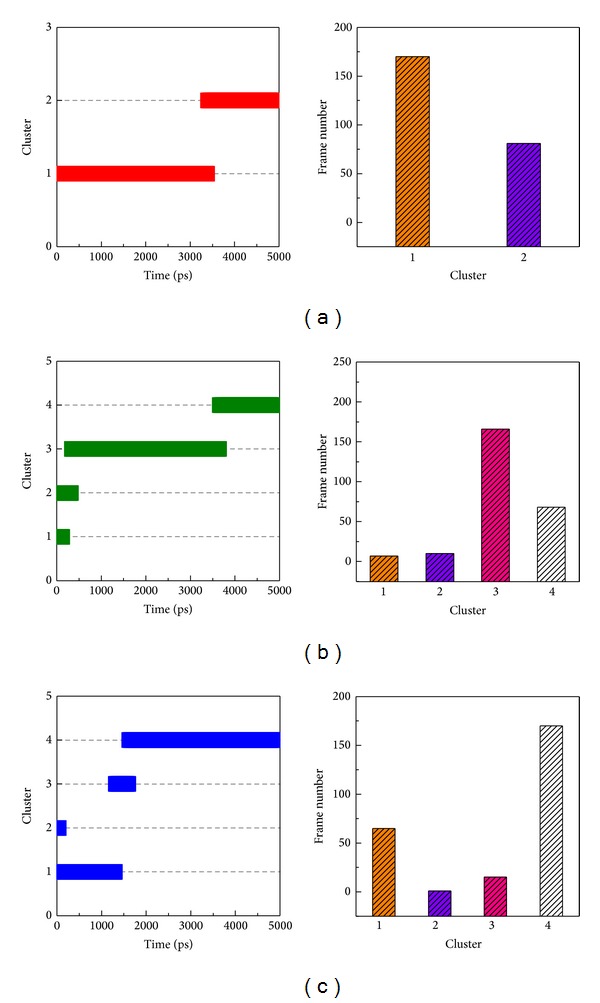
Clustering analyses of protein conformations: (a) Solapalmitine, (b) Isodesacetyluvaricin, and (c) Budmunchiamine L5 with simulation times of 5000 ps.

**Figure 10:**
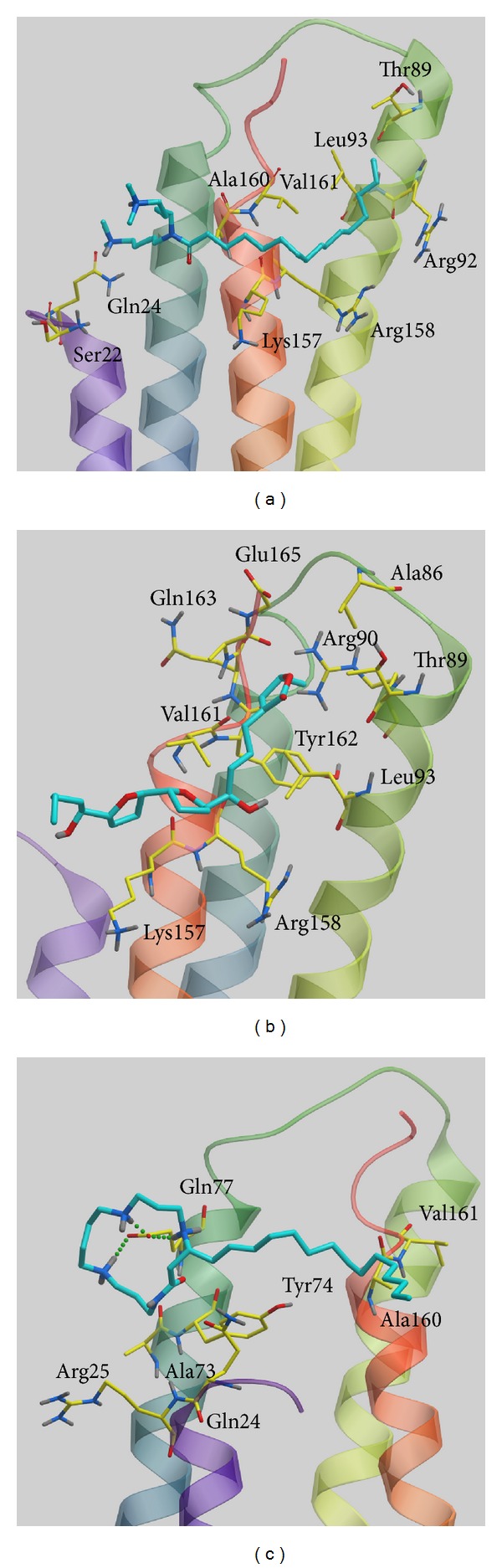
The middle structure from each final clustering group with docked ligand: (a) Solapalmitine, (b) Isodesacetyluvaricin, and (c) Budmunchiamine L5. The small molecular and amino acids are colored green and yellow, respectively.

**Figure 11:**
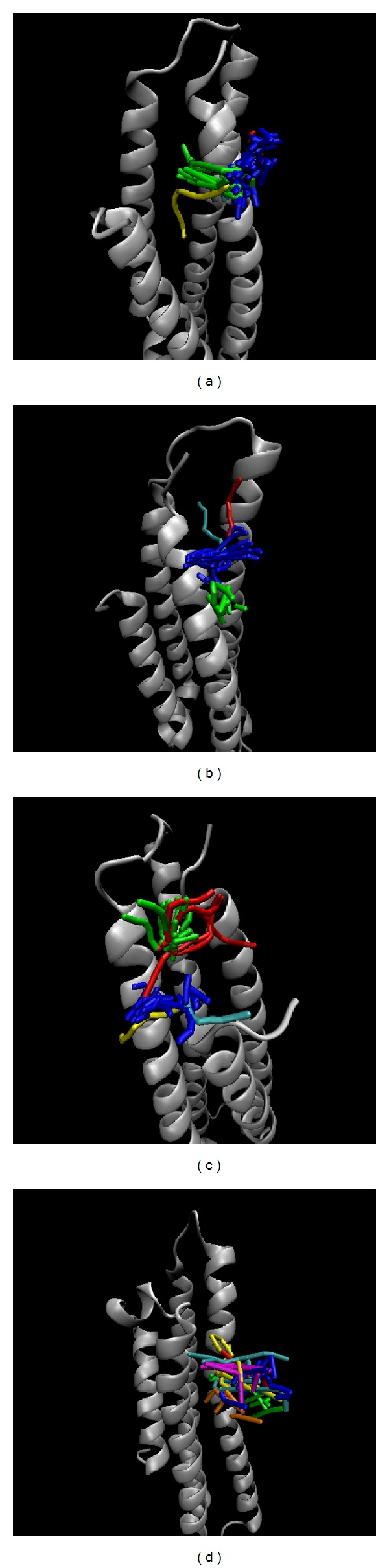
Ligand pathway prediction form protein conformations: (a) Solapalmitine, (b) Isodesacetyluvaricin, and (c) Budmunchiamine L5 and (d) Apo form of APOE4 with simulation times of 5000 ps.

A cluster algorithm was employed to select the most stable conformation over all simulation times. All MD snapshots with docked ligands were grouped into two or four individual clusters (Figure 9) with each cluster including similar conformations. The middle structure was chosen from each late group as a standard snapshot for the binding analysis of the top three TCM compounds: Solapalmitine (4040 ps), Isodesacetyluvaricin (4240 ps), and Budmunchiamine L5 (4340 ps) (Table 2). In the binding analysis from the standard snapshot (Figure 10), we found that Budmunchiamine L5 exhibited H-bond interaction with Glu77, but the H-bonds disappeared between Isodesacetyluvaricin and Arg158. Solapalmitine continued to have no H-bond interactions between residues. The data shows that Glu77 is a crucial residue for Budmunchiamine L5 binding. We also employed CAVER 3.0 software [71] to analyze the migrated ligand tunnels in ApoE4 (Figure 11), and the ligand pathway analysis was used to predict in previous studies [40]. The prediction of ApoE4 showed lower number of channels than Apo form of ApoE4; the results illustrated that the TCM candidates could form stable binding conformation to interact with ApoE4 with all simulation time.

## 4. Conclusion

Solapalmitine is the top candidate by LibDock score but displays significantly increasing MSD values due to unstable binding with ApoE4 over 5000 ps simulation time. Isodesacetyluvaricin has H-bond with Arg158; unfortunately, the H-bond is missing during MD simulation. The LibDock score of Budmunchiamine L5 is not the highest score from the TCM database screening; however, the dynamics simulation shows that the docked ligand complex of ApoE4 is stable. In snapshot analysis, Budmunchiamine L5 still forms an H-bond with Glu77; the binding pose is the same as the initial docking pose, suggesting that Budmunchiamine L5 does not change over all simulation times and stably binds to ApoE4. In terms of ADMET analysis, Budmunchiamine L5 has good absorption, BBB penetration, and less toxicity in the human liver and may therefore be regarded as a safe lead drug for designing a novel ApoE4 inhibitor for AD therapy.

## Figures and Tables

**Table 1:** ADMET prediction of top ten TCM compounds from docking results.

Name	LibDock score	^ a^Absorption	^ b^BBB Level	^ c^CYP2D6	^ d^Hepatotoxicity
Solapalmitine	**118.404**	**0**	**0**	**0**	**0**
Isodesacetyluvaricin	**116.555**	**0**	**2**	**0**	**0**
Budmunchiamine L5	**116.167**	**0**	**2**	**0**	**0**
Hemiariensin	115.334	0	2	0	0
Niranthin	112.802	0	1	0	0
Platyphyllonol	112.212	0	2	0	0
Triptofordin B1	111.954	0	2	0	0
Aglaiduline	111.306	0	2	0	0
Aurantiamide	110.823	0	2	0	0
Lobelanidine	108.696	0	2	0	0

^a^Absorption: good absorption = 0; moderate absorption = 1; low absorption = 2; ^b^BBB level (blood brain barrier): very high penetration = 0; high penetration = 1; medium penetration = 2; low penetration = 3; undefined penetration = 4.

^c^CYP2D6: noninhibitor = 0, inhibitor = 1.

^d^Hepatotoxicity: noninhibitor = 0, inhibitor = 1.

**Table 2:** Time of middle structure in each cluster from all MD simulation times.

Cluster	Time of middle flame (ps)		
	Solapalmitine	Isodesacetyluvaricin	Budmunchiamine L5
1	1540	60	640
2	4040	280	40
3	—	1920	1480
4	—	4240	4340
